# The Positive Feedback Loop of Hypoxia-Inducible Factor-1*α*/miR-295/Factor Inhibiting Hypoxia-Inducible Factor-1 in Hyperuricemic Nephropathy

**DOI:** 10.34067/KID.0000001069

**Published:** 2025-12-23

**Authors:** Jiachang Li, Yuhan Ma, Yanni Wang, Yizhi Chen, JiaLi Wei

**Affiliations:** 1 Department of Nephrology, Hainan Affiliated Hospital of Hainan Medical University (Hainan General Hospital), Haikou, Hainan, China; 2 PSB Academy, Singapore; 3 Department of Nephrology, Hainan Hospital of Chinese PLA General Hospital, Academician Chen Xiangmei of Hainan Province Kidney Diseases Research Team Innovation Center, Sanya, China

**Keywords:** interstitial fibrosis, nephropathy, renal fibrosis

## Abstract

**Key Points:**

- Hypoxia-inducible factor-1*α*–mediated induction of miR-295 in renal tubular cells in hyperuricemic nephropathy.
- This study reveals a hypoxia-inducible factor-1*α*/miR-295/factor inhibiting hypoxia-inducible factor-1 positive feedback loop that regulates tubular damage and fibrosis in hyperuricemic nephropathy.

**Background:**

Hyperuricemia is a common metabolic disorder and a risk factor for multiple diseases, including CKD. Hyperuricemic nephropathy (HN) affects many individuals with hyperuricemia, yet its molecular mechanisms are not fully understood, and effective treatments are lacking.

**Methods:**

*In vitro*, human tubular epithelial cells (HK-2) were exposed to uric acid for 36 hours, followed by transfection with microRNA mimic or factor inhibiting hypoxia-inducible factor-1 (FIH-1) siRNA. *In vivo*, HN was induced in mice using potassium oxonate and adenine for 2 weeks. miR-295 mimic or anti-miR-295 was administered through tail vein injection, and mice were euthanized for analysis.

**Results:**

We demonstrated a significant increase of miR-295 in renal tubular cells in HN mice. Hyperuricemia led to the activation of hypoxia inducible factor-1*α* (HIF-1*α*), and inhibition of HIF-1*α* by YC-1 (a HIF-1*α* inhibitor) prevented the increase of miR-295. Chromatin immunoprecipitation assay further verified HIF-1*α* binding to the miR-295 gene promoter directly. Functionally, inhibition of miR-295 led to increased cell death and tubulointerstitial fibrosis in HN mice, whereas supplementation of miR-295 mimic had kidney-protective effects in this model. miR-295 suppressed the expression of FIH-1) in both *in vitro* and *in vivo* models of HN. Luciferase microRNA target reporter assay further verified FIH-1 as a direct target of miR-295. In addition, knockdown of FIH-1 inhibits tubular cell apoptosis and profibrotic cytokines production in HK2 cells during uric acid treatment.

**Conclusions:**

This study reveals a HIF-1*α*/miR-295/FIH-1 positive feedback loop that regulates tubular damage and fibrosis in HN.

## Visual Abstract

**Figure:**
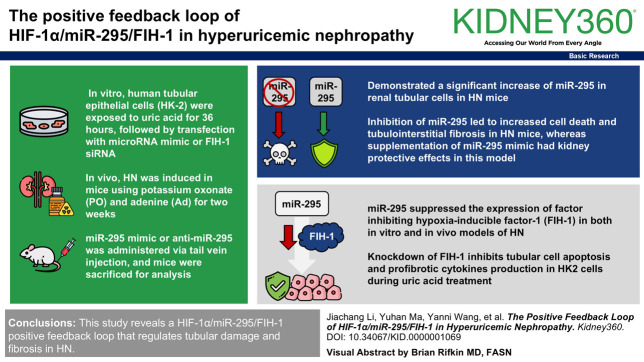

## Introduction

With the rapid development of the world economic and the improvement of lifestyle, the prevalence of hyperuricemia is remarkably increasing all over the world.^1^ Uric acid (UA) is the final product of purine metabolism in human body, and 70% of UA is excreted through kidneys. Once UA concentration is beyond the physiological range, it will cause a variety of pathological responses, such as oxidative stress, mitochondrial dysfunction, apoptosis, and inflammation.^2,3^ An increasing number of those studied have indicated that chronic UA injury to the kidney is sufficient to trigger renal tubular damage, interstitial fibrosis, glomerulosclerosis, and urate crystal deposition, leading to hyperuricemic nephropathy (HN).^4–6^ Epidemiologically, HN affects many patients with hyperuricemia; however, the specific molecular mechanism of HN remains unclear, and there is still a lack of effective therapies.

Tubulointerstitial fibrosis is believed to be the primary pathogenesis for progressive HN.^4,5,7^ In fact, renal fibrosis is the final common pathway and the histological manifestation of CKD.^8^ Fibrosis is characterized by loss of capillary networks, accumulation of fibrillary collagens, activated myofibroblasts, and inflammatory cells.^9^ Recently, in renal fibrosis, studies have indicated that tubular epithelial cells are lost because of cell death and the remaining cells dedifferentiate, leading to reduced expression of characteristic epithelial markers and increased expression of mesenchymal markers.^10^ These disordered proximal tubules act as a driving force for interstitial fibrosis through activation of autocrine and paracrine signals. A long-term of UA exposure causes tubular epithelial cell injury and a maladaptive renal repair with the prolonged cell cycle G2/M arrest.^11,12^ Thus, the proximal tubule may be a new therapeutic target for patients with HN. In fact, studies have shown that tubular injury was a critical component of the early course of renal fibrosis and also suggested to contribute in a primary way, rather than a secondary manner, to the development of early renal fibrosis.^13^

Hypoxia inducible factor 1*α* (HIF-1*α*) is a key regulatory molecule in mammals under hypoxic conditions.^14^ In addition, it has been indicated that HIF-1*α* is involved in CKD progression caused by various stimuli, such as ischemia–reperfusion, aristolochic acid, and unilateral ureteral occlusion.^14–16^ In fact, there are three subtypes of HIFs named HIF-1, HIF-2, and HIF-3 because of the labile subunit HIF-*α* (HIF-1*α*, HIF-2*α*, or HIF-3*α*). HIF-1*α* is ubiquitously expressed in organs of most cell types, whereas HIF-2*α* expression is tissue limited and detected particularly in highly vascularized tissues and organs.^17^ In the kidney, HIF-1*α* is found in most renal epithelial cells, while HIF-2*α* is mainly expressed in renal interstitial fibroblast-like cells and endothelial cells.^17^ Although HIF-1*α* has been implicated in the pathogenesis of renal fibrosis, it is still controversial whether HIF-1*α* is protective or pathogenic.^16^

miRNAs are small noncoding RNA molecules composed of approximately 22 nucleotides.^18^ Until now, 39,000 miRNAs have been identified in humans, 365 of which are present on the renal cortex.^19^ In mammalian cells, miRNAs repress gene expression mainly by binding to the 3′-untranslated regions (UTR) of their target gene mRNA, thereby blocking their translation. mRNAs are known to regulate animal development, physiology, and the pathogenesis of various diseases, including renal fibrosis.^18,20^ In renal fibrosis, studies have identified a variety of profibrotic and antifibrotic miRNAs.^21^ For example, Lan *et al*. demonstrated that miR-433 acted as a profibrotic factor. They showed that unilateral ureteral obstruction in mice was associated with miR-433 induction, which targeted Azin1 for the activation of the transforming growth factor-*β* (TGF-*β*)/Smad3 signaling pathway for renal fibrosis.^22^ However, in other studies, miR-29 family members, including miR-29a, miR-29b, and miR-29c, have been reported as antifibrotic factors in CKD, such as in diabetic nephropathy, FSGS, membranous nephropathy, and IgA nephropathy.^21^ However, there is little research on the role of miRNAs in HN.

In this study, we have demonstrated that HIF-1*α* is activated to promote miR-295 expression in renal tubular cells in HN. Functionally, inhibition of miR-295 led to increased cell death and tubulointerstitial fibrosis after adenine (Ad) and potassium oxalate treatment in mice, whereas supplementation of miR-295 mimic had kidney protective effects in this model. Interestingly, we have further identified factor inhibiting hypoxia-inducible factor-1 (FIH-1) as a direct target of miR-295. Thus, induction of miR-295 by HIF-1*α* in HN results in the suppression of FIH-1, further enhancing the HIF-1*α* activation, providing a positive feedback mechanism to contain HIF-1*α* activation and associated renal fibrosis.

## Methods

### Chemicals and Reagents

Antibodies in the present study were from the following sources: Anti-FIH-1(ab92498), anti-fibronectin (Fn, ab2413), anti-*α*-smooth muscle actin (SMA) (ab5694), anti-HIF-1*α* (ab179483) were from Abcam (Cambridge, United Kingdom). Anti-cleaved caspase-3(9664) and anti-glyceraldehyde-3-phosphate dehydrogenase (GAPDH) (5174) were from Cell Signaling Technology (Boston, MA). All secondary antibodies for immunoblotting analysis were from Thermo Fisher Scientific (Waltham, MA). FITC/CY3-conjugated goat anti-rabbit IgG was from Abcam. Special reagents: Potassium oxonate (PO; purity ≥98%) and Ad (purity ≥98%) were purchased from Aladdin., Ltd (Shanghai, China). UA was obtained from Sigma (St. Louis, MO). The kits for analysis of UA, creatinine (CRE), and BUN were acquired from Dibao medical (Guangzhou, China). The Digoxigenin-labeled mmu-miR-295 locked nucleic acid (LNA) probe and Fluorescence *in situ* hybridization (FISH) Kit were from Servicebio (Wuhan, China). miR-295 mimic, anti-miR-295 LNA, and FIH-1 siRNA were from Ruibo (Guangzhou, China). YC-1 was from Selleck Chemicals (Houston). The chromatin immunoprecipitation assay kit (ab500) was from Abcam.

### Animals and HN Induction

Eight-week-old male C57BL/6 mice were purchased from the Slaccas Animal Laboratory (Haikou, China) and housed under controlled environmental conditions (temperature of 22℃, 12 -hour darkness period). The protocol was approved by the Institutional Animal Care and Use Committee. HN was induced by PO intraperitoneally and Ad was orally administered for 2 weeks. Briefly, except for the control group, each group was intraperitoneally administered PO (350 mg/kg per day) and orally administered with Ad (70 mg/kg per day) to induce HN at 8:30 am for 14 consecutive days according to previous reports with few modifications.^23^ Control mice were treated with normal saline. YC-1 (a selective HIF-1*α* inhibitors, 50 mg/kg, Selleck) was given intraperitoneally on the day of PO injection and continued until the mice were euthanized. In some experiments, miR-295 mimic (3 mg/kg), anti-miR-295 LNA (6  mg/kg), or negative control (NC) oligonucleotide LNA were delivered to mice through tail vein injection every 2 days after PO injection. At the end of 21st day, after blood and urine samples were collected from the retro-orbital sinus, the animals were euthanized, and the kidneys were isolated for histological and molecular biological analysis.^23^

### Cell Culture and Treatments

Human tubular epithelial cells (HK-2) were cultured in a 1:1 mixture of DMEM and F-12 containing 10% FBS, 1% penicillin-streptomycin in an atmosphere of 5% CO_2_ and 95% air at 37°C. Before starting the formal experiments, we passed the primary cells for three generations to obtain a stable phenotype. The HK-2 cells were starved for 24 hours in DMEM medium containing 0.5% FBS and then exposed to UA (800 *μ*M) for 36 hours. After stimulation for 36 hours, cells were harvested for further analysis. In some experiments, 200 nM microRNA mimic, FIH-1 siRNA (40 nM), 50 nM HIF-1*α* plasmid, or NC oligonucleotides were transfected into HK2 cells with lipofectamine 2000, following the manufacturer's instructions. All the *in vitro* experiments were repeated no less than three times.^24^

### Renal Histology and Terminal Deoxynucleotidyl Transferase Deoxyuridine Triphosphate Nick-End Labeling Assay

Kidney tissues were fixed with 4% paraformaldehyde and embedded in paraffin. Kidney tissue sections of 4 *μ*m were then prepared. Hematoxylin–eosin (H&E) and Masson staining was conducted to analyze renal histology. terminal deoxynucleotidyl transferase deoxyuridine triphosphate nick-end labeling (TUNEL) assay in kidney tissue sections or cultured cells was performed as previously described.^18^ The slides were imaged with fluorescent microscopy and for the TUNEL-positive cells, we analyzed 10 high-power fields of view per sample, with five samples included in each group.

### Immunoblot Analysis

The total protein was extracted from renal tissues or cultured cells through radio-immunoprecipitation assay lysis buffer with protease inhibitors and separated by 12% SDS PAGE. After that, protein samples were transferred to a polyvinylidene fluoride membrane. Then, the membranes were blocked with 5% skim milk and probed overnight at 4°C with primary antibodies against HIF-1*α* (1:1000), cleaved-caspase 3 (1:1000), *α*-SMA (1:1000), fibronectin (1:1000), FIH-1 (1:1000), and GAPDH (1:3000). After washing, horseradish peroxidase-conjugated secondary antibodies (goat anti-rabbit IgG 1:5000) were applied for 1 h at room temperature. The protein membrane was finally detected by an ECL Detection system (Amersham Pharmacia Biotech, Little Chalfont, United Kingdom). For densitometric analysis of Western blot bands, Image J software was used to measure the gray intensity of target proteins (HIF-1*α*, cleaved-caspase 3, *α*-SMA, Fibronectin and FIH-1) and the internal reference protein GAPDH. The relative expression levels of target proteins were calculated as the ratio of target protein gray intensity to GAPDH gray intensity. For normalization, the ratio of HIF-1*α*/GAPDH, cleaved-caspase 3/GAPDH, *α*-SMA/GAPDH, and fibronectin/GAPDH in the control group was set as 1.0, and the values of other experimental groups were normalized relative to this baseline.^25^

### Immunofluorescence

Paraffin-embedded kidney sections were sequentially subjected to deparaffinization, hydration, and antigen retrieval by incubation with 0.1 M sodium citrate, pH 6.0, at 100°C. Kidney sections or fixed cells were then permeabilized with 0.1% Triton ×-100 and incubated in blocking buffer. The specimens were sequentially incubated with related primary antibodies at 4°C overnight, FITC-conjugated or rhodamine-conjugated secondary antibodies for 1 hour at room temperature, and 4′,6-diamidino-2-phenylindole (MilliporeSigma, D9542). Then, the slides were examined with a fluorescence microscope, and the representative figures were exhibited.

### Luciferase microRNA Target Reporter Assay

The 3′-UTR of the mouse FIH-1 gene inserted into the 3′-UTR of the luciferase gene in the pMIR-REPORT luciferase plasmid. The plasmids with or without the insert were cotransfected with pMIR-REPORT *β*-gal control plasmid and 200  nM miR-295 mimics into HEK293 cells. One day after the transfection, the lysate was collected in reporter lysis buffer from the Luciferase Assay System (Promega, Madison, WI). The luciferase activity was normalized with *β*-galactosidase activity. The ratio of the normalized value between miR-295 and the NC group was used for comparison.

### Chromatin Immunoprecipitation Assay

The cells were fixed with 1% formaldehyde and then neutralized with glycine. The cell samples were then sonicated to shear the DNA. After that, the supernatants containing DNA were collected. Equal amounts of DNA from different samples were incubated with equal amount of anti-HIF-1*α* antibody. The resultant immunoprecipitate was subjected to qPCR amplification of putative HIF-1*α* binding sequences using specifically designed primers. The value of qPCR was normalized with input DNA for comparison.

### FISH

FISH was performed according to the manufacturer's instructions. Briefly, kidneys were harvested from control and HN mice to prepare 4-micron paraffin section. The sections were treated with 20  *μ*g/ml proteinase K for permeabilization and then incubated with prehybridization solution at 78°C for 1 hour. We then removed prehybridization solution and added digoxigenin-labeled mmu-miR-295 LNA probe over night at 37°C. At the second day, after wash, BSA was added for blocking. Then the anti-digoxigenin-horseradish peroxidase was used at 37°C for 1 hour. CY3-TSA and 4′,6-diamidino-2-phenylindole assay were used to indicate the positive areas and cell nucleus, respectively. In addition, to better determine the expression location of miR-295 in the kidney, we performed double staining of miR-295 with the proximal tubule marker *Lotus tetragonolobus* lectin. This costaining strategy allows clear visualization of whether miR-295 is expressed in proximal tubule cells, thereby clarifying its spatial distribution in renal tissue. The images were acquired from a fluorescence microscope, and the representative figures were exhibited.

### qPCR

Total RNA was isolated from the kidney tissues/cultured cells using TRIzol (Invitrogen; MA). For qPCR analysis of miRNAs, total RNAs from each sample were reversely transcribed into cDNA by using the microRNA Reverse Transcription kit (Applied Biosystems), and qPCR analysis of target miRNA was performed by using the TaqMan microRNA assay kit (Applied Biosystems). For qPCR analysis of mRNAs, total RNAs from each sample were reversely transcribed into cDNA by using the M-MLV Reverse Transcriptase cDNA Synthesis Kit (TaKaRa Bio US). qPCR was performed by using the SYBR Premix Ex Taq TM II (TaKaRa Bio US). All PCR data were analyzed by the LightCycler 96 SW 1.1 software, and each sample was shown as 2-ΔΔCt values.

### Statistical Analysis

All data, expressed as the mean±SD, were evaluated using SPSS 22.0 (IBM, NY) and GraphPad Prism 10.0 (GraphPad Software, La Jolla, CA). Student *t* test was used to show the significant difference between two groups, and ANOVA analysis was used for multigroup difference analysis. At *P* < 0.05, it was of statistical significance.

## Results

### miR-295 is Induced in Renal Tubules in HN Mice

**Figure 1:**
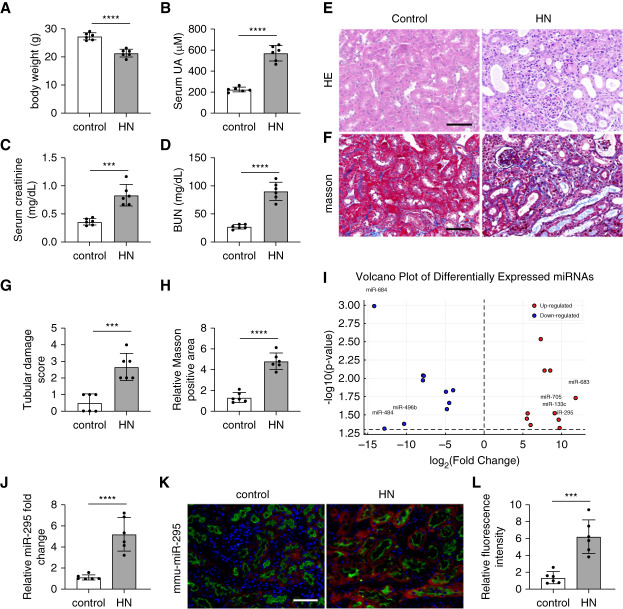
**miR-295 is induced in renal tubules in HN mice.** HN was induced by PO intraperitoneally and Ad orally administered for 2 weeks. Except for the control group, each group was intraperitoneally administered PO (350 mg/kg per day) and orally administered with Ad (70 mg/kg per day) to induce HN at 8:30 am for 14 consecutive days. Control mice were treated with normal saline. At the end of 21st day, the animals were euthanized. (A) Body weight changes in control and HN mice; (B) serum UA concentrations in each group; (C) serum CRE levels; (D) BUN levels; (E) representative images of HE staining, scale bar: 50 *μ*m; (F) representative images of Masson staining, scale bar: 50 *μ*m; (G) quantitative analysis of tubular injury score in different experimental groups; (H) quantification of Masson's trichrome–positive fibrotic area in kidney sections; (I) volcano plot of microRNA expression profiled by microarray in HN. Differentially expressed miRNAs were identified using data obtained from three biological replicates per experimental group (*n*=3 mice per group, total six samples). Statistical significance was determined using the criteria of |log_2_ fold change| >1.5 and *P* < 0.05. These thresholds were applied to ensure robust identification of meaningful expression differences. (J) qPCR analysis of miR-295 in mouse kidneys. The level of miR-295 was normalized to the level of U6 (internal loading control) of the same samples to determine the ratio with the ratio of control mice arbitrarily set as 1. (K) *In situ* hybridization showing miR-295 increase in kidney tissues. Representative images of double immunostaining with miR-295 and proximal tubule marker, LTL, scale bar: 50 *μ*m. (L) Quantitative analysis of fluorescence intensity. All the values are expressed as mean±SD (*n*=6), *P  < 0.05, **P  < 0.01, ***P  < 0.001, ****P < 0.0001. Ad, adenine; CRE, creatinine; HE, hematoxylin–eosin; HN, hyperuricemic nephropathy; LTL, *Lotus tetragonolobus* lectin; PO, potassium oxonate; UA, uric acid

To identify specific miRNAs involved in the pathogenesis of HN, we initially tested a mouse model. As shown in Figure 1, the body weight of the HN mice decreased compared with the control mice (Figure 1A), while their serum UA levels significantly increased (Figure 1B). In addition, both serum CRE and BUN levels were elevated in the HN mice on day 21 compared with the control mice (Figure 1, C and D). Histological analysis using H&E and Masson staining revealed significant tubular injury and tubulointerstitial fibrosis in the HN mice (Figure 1, E–H), confirming the successful establishment of the HN mouse model. We then collected kidney cortical tissues for microarray analysis of miRNA expression, identifying several miRNAs with altered expression, including miR-295 (Figure 1I). TaqMan real-time PCR further confirmed the increase of miR-295 in the kidneys of HN mice compared with control mice (Figure 1J). *In situ* hybridization analysis and double immunofluorescence staining using the proximal tubule marker (*L*. *tetragonolobus* lectin) demonstrated miR-295 induction after PA treatment in renal proximal tubules (Figure 1, K and L). Importantly, our experiments also further revealed that miR-295 may be upregulated only in HN, but not in other types of CKD, such as diabetic nephropathy (Supplemental Figure 1).

### HIF-1*α* Mediates the Induction of miR-295 in HN

**Figure 2:**
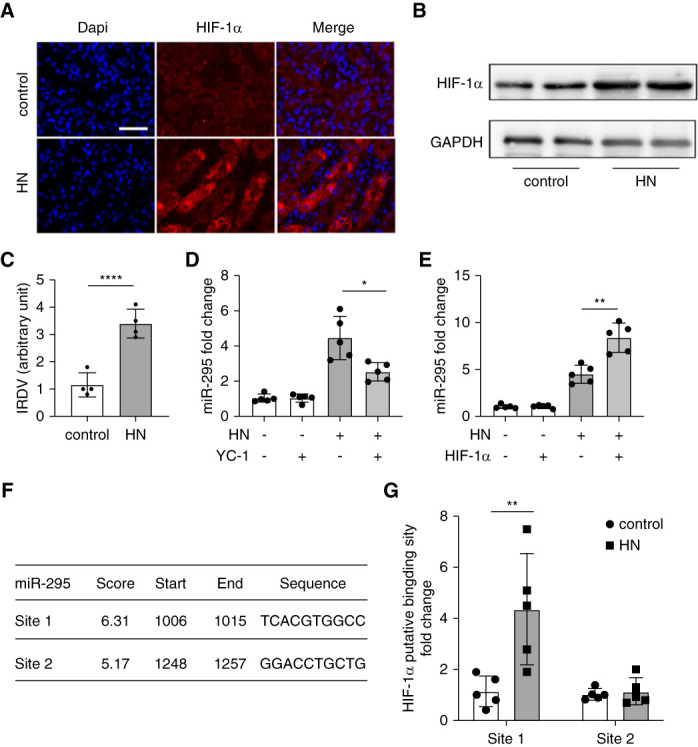
**HIF-1*α* mediates the induction of miR-295 in HN.** HN was induced by PO intraperitoneally and Ad orally administered for 2 weeks. Except for the control group, each group was intraperitoneally administered PO (350 mg/kg per day) and orally administered with Ad (70 mg/kg per day) to induce HN at 8:30 am for 14 consecutive days. Control mice were treated with normal saline. At the end of 21st day, the animals were euthanized. YC-1 (a selective HIF-1*α* inhibitors, 50 mg/kg, Selleck) was given by intraperitoneally on the day of PO injection and continued until the mice were euthanized. (A) Representative images of immunofluorescence staining of HIF-1*α*, scale bar: 50 *μ*m; (B) expressions of HIF-1*α* was detected by Western blot; GAPDH was used as internal control; (C) quantification analysis of IRDV of HIF-1*α*, data are expressed as mean±SD (*n*=4), *P < 0.05, **P< 0.01, ***P < 0.001, ****P < 0.0001; (D) qPCR analysis showing the induction of miR-295 in HN is partly prevented by YC-1. The levels of miR-295 were normalized to the levels of U6 of the same samples to determine the ratios. Data are expressed as mean ± SD (*n*=5), *P < 0.05, **P <0.01, ***P <0.001, ****P < 0.0001; (E) qPCR analysis showing the induction of miR-295 in HN was further enhanced by HIF-1*α* transfection. HK2 cells were transfected with 50 nM HIF-1*α* plasmid and then treated with UA (800 *μ*M) for 36 hours. The levels of miR-295 were normalized to the levels of U6 of the same samples to determine the ratios. Data are expressed as mean±SD (*n*=5), **P < 0.05, **P < 0.01, ***P < 0.001, ****P < 0.0001; (F) predicted HIF-1*α* binding site in human miR-295 gene promoter. (G) ChIP analysis of HIF-1*α* binding in miR-295 promoter. HK-2 cells were treated with or without UA (800 *μ*M) for 36 hours to collect the chromatin for immunoprecipitation with a specific anti-HIF-1*α* antibody. The immunoprecipitated samples were subjected to qPCR analysis of miR-295 promoter sequence. Quantitative data are expressed as mean±SD (*n*=5), *P < 0.05, **P < 0.01, ***P < 0.001, ****P < 0.0001. ChIP, chromatin immunoprecipitation; DAPI, 4′,6-diamidino-2-phenylindole; GAPDH, glyceraldehyde-3-phosphate dehydrogenase; HIF-1*α*, hypoxia-inducible factor-1*α*; IRDV, integrated relative densitometric value.

As mentioned, HIF-1*α* plays a crucial role in tubulointerstitial fibrosis in CKD by influencing oxidative stress, mitochondrial dysfunction, apoptosis, and inflammation.^26,27^ Thus, we hypothesized that HIF-1*α* might be involved in miR-295 induction in HN. Immunofluorescence showed a significant increase in renal tubular cells with HIF-1*α*-positive nuclei or cytoplasm (Figure 2A). Immunoblot analysis further confirmed HIF-1*α* induction in HN (Figure 2, B and C). To investigate HIF-1*α*′s role in miR-295 induction, we tested YC-1, a specific HIF-1*α* inhibitor. As shown in Figure 2D, YC-1 partially inhibited miR-295 induction after PO and Ad treatment. To further clarify the relationship between HIF-1*α* and miR-295, we performed additional *in vitro* experiments. Results showed that after transfection of HK2 cells with the HIF-1*α* plasmid followed by UA treatment, the expression of miR-295 was further upregulated (Figure 2E). This finding further suggests that the expression of miR-295 is dependent on HIF-1*α*. Bioinformatics analysis using the JASPAR Database (http://jaspar.genereg.net/) identified two potential HIF-1*α* binding sites in the miR-295 gene promoter, labeled as sites 1 and 2 (Figure 2F). Chromatin immunoprecipitation assay demonstrated increased binding of HIF-1*α* to site 1 in the miR-295 gene promoter in HN (Figure 2G). These findings suggest that HIF-1*α* may regulate miR-295 induction in HN by directly influencing gene transcription.

### miR-295 Attenuates Kidney Injury and Renal Fibrosis in HN

**Figure 3:**
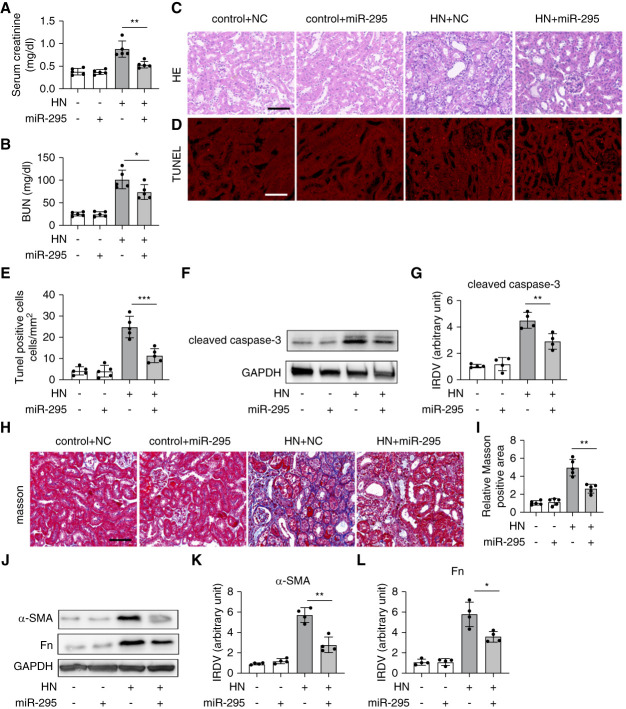
**miR-295 attenuates kidney injury and renal fibrosis in HN.** HN was induced by PO intraperitoneally and Ad orally administered for 2 weeks. Except for the control group, each group was intraperitoneally administered PO (350 mg/kg per day) and orally administered with Ad (70 mg/kg per day) to induce HN at 8:30 am for 14 consecutive days. Control mice were treated with normal saline. At the end of 21st day, the animals were euthanized. miR-295 mimic (3 mg/kg) or NC oligonucleotide LNA were delivered to mice through tail vein injection every 2 days after PO injection. (A) Serum CRE; (B) BUN levels; (C) representative images of H&E staining, scale bar: 50 *μ*m; (D) representative images of TUNEL staining, scale bar: 50 *μ*m; (E) quantification of TUNEL-positive cells. (F) Immunoblot analysis of cleaved caspase-3, GAPDH was used as loading control; (G) quantification analysis of the related band intensity of cleaved caspase-3; IRDV. (H) Representative images of Masson staining, scale bar: 50 *μ*m; (I) quantification of Masson's trichrome–positive fibrotic area in kidney sections; (J) immunoblot analysis of Fn and *α*-SMA, GAPDH was used as loading control; (K and L) quantification analysis of the related band intensity of Fn and *α*-SMA. All the quantitative data are expressed as mean±SD, *P < 0.05, **P < 0.01, ***P < 0.001, ****P < 0.0001. Fn, fibronectin; LNA, locked nucleic acid; NC, negative control; SMA, smooth muscle actin; TUNEL, terminal deoxynucleotidyl transferase deoxyuridine triphosphate nick-end labeling.

To clarify the role of miR-295 in HN, we first assessed the impact of *in vivo* transfection of miR-295 mimics. We confirmed that this transfection led to a sustained increase of miR-295 in kidney tissues (Supplemental Figure 2). In control mice, the miR-295 mimic did not cause any functional or structural kidney damage, but it significantly reduced serum CRE and BUN levels and lessened kidney tissue damage in HN mice (Figure 3, A–C). In addition, TUNEL assay revealed that miR-295 mimics reduced renal tubular cell death in HN mice (Figure 3, D and E). Immunoblot analysis showed that *in vivo* delivery of the miR-295 mimic significantly lowered cleaved caspase-3 levels in HN mice (Figure 3, F and G). For renal fibrosis, Masson staining indicated that miR-295 mimic delivery in HN mice significantly decreased collagen expression (Figure 3, H and I). Further immunoblot analysis demonstrated that both Fn and *α*-SMA levels were reduced in the HN+miR-295 group compared with the HN+NC group (Figure 3, J–L). These results suggest that miR-295 plays a protective role in HN.

### Inhibition of miR-295 Exaggerates Kidney Injury and Renal Fibrosis in HN

**Figure 4:**
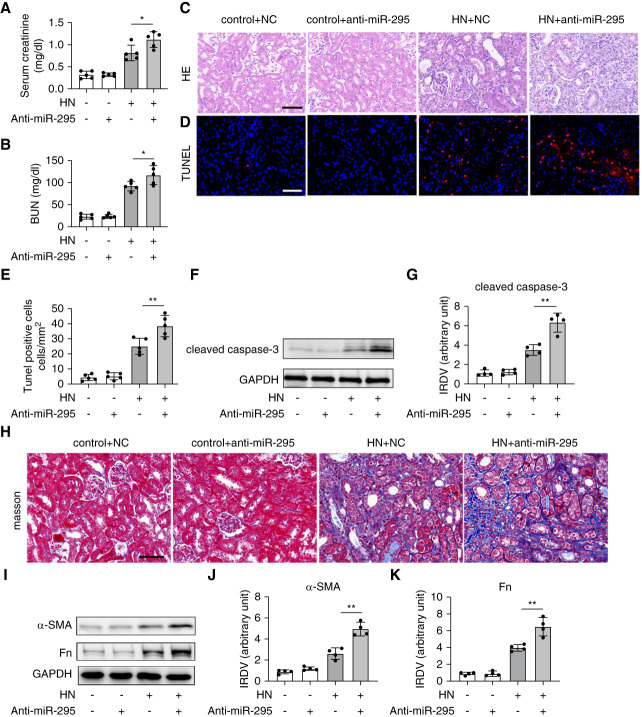
**Inhibition of miR-295 exaggerates kidney injury and renal fibrosis in HN**. HN was induced by PO intraperitoneally and Ad orally administered for 2 weeks. Except for the control group, each group was intraperitoneally administered PO (350 mg/kg per day) and orally administered with Ad (70 mg/kg per day) to induce HN at 8:30 am for 14 consecutive days. Control mice were treated with normal saline. At the end of 21st day, the animals were euthanized. Anti-miR-295 LNA (6  mg/kg) or NC oligonucleotide LNA were delivered to mice through tail vein injection every 2 days after PO injection. (A) Serum CRE; (B) BUN levels; (C) representative images of H&E staining, scale bar: 50 *μ*m; (D) representative images of TUNEL staining, scale bar: 50 *μ*m; (E) quantification of TUNEL-positive cells. (F) Immunoblot analysis of cleaved caspase-3, GAPDH was used as loading control; (G) quantification analysis of the related band intensity of cleaved caspase-3, IRDV; (H) representative images of Masson staining, scale bar: 50 *μ*m; (I) immunoblot analysis of Fn and α-SMA, GAPDH was used as loading control; (J–K) quantification analysis of the related band intensity of Fn and *α*-SMA. All the quantitative data are expressed as mean±SD, *P < 0.05, **P < 0.01, ***P < 0.001, ****P < 0.0001.

To further investigate the role of miR-295 in HN, we examined the effects of anti-miR-295. At first, we confirmed that we successfully transfected anti-miR-295 LNA into mice *in vivo* (Supplemental Figure 3). Like the miR-295 mimic, anti-miR-295 did not cause any functional or structural kidney damage in control mice, but it significantly increased serum CRE and BUN levels and worsened kidney structural damage in HN mice (Figure 4, A–C). In addition, anti-miR-295 promoted renal apoptosis in HN mouse models, as evidenced by TUNEL staining (Figure 4, D and E) and cleaved caspase-3 immunoblotting (Figure 4, F and G). Anti-miR-295 also exacerbated renal fibrosis in HN mice, as shown by Masson staining and immunoblot analysis of Fn and *α*-SMA (Figure 4, H–K). These findings further support the protective role of miR-295 in HN.

### FIH-1 is a Downstream Target of miR-295 in HN

**Figure 5:**
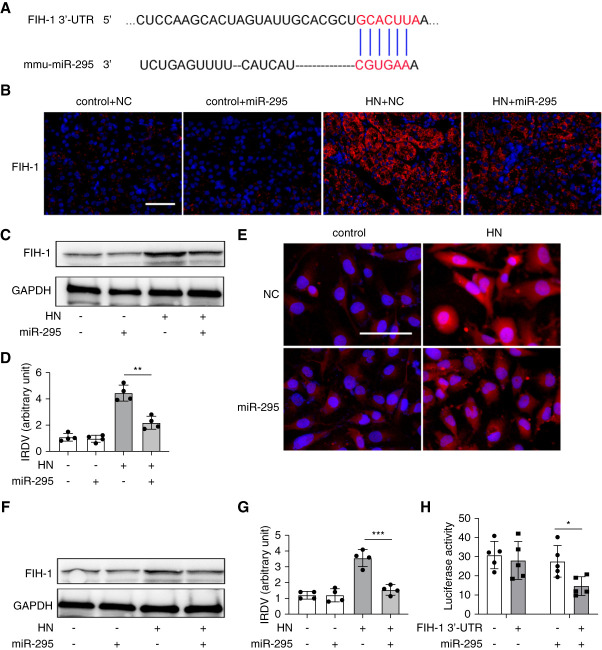
**FIH-1 is a downstream target of miR-295 in HN.** (A) Conserved miR-295 target sequence in the 3′-UTR of FIH-1 mRNA. (B) Immunofluorescence showing the repressive effect of miR-295 on FIH-1 expression, scale bar: 50 *μ*m. miR-295 mimic (3 mg/kg) or NC oligonucleotide LNA were delivered to mice through tail vein injection every 2 days after PO injection. At the end of 21st day, the animals were euthanized. (C) Immunoblot analysis showing the repressive effect of miR-295 on FIH-1 expression. Mice were subjected to the same treatment as in B to collect kidney tissues for immunoblot analysis. GAPDH was used as internal control. (D) Quantification analysis of the related band intensity of FIH-1. IRDV (E) Immunofluorescence showing the repressive effect of miR-295 on FIH-1 expression, scale bar: 50 *μ*m. HK2 cells were transfected with 200 nM miR-295 mimic or NC for 24 hours and then treated with or without UA (800 *μ*M) for 36 hours. (F) Immunoblot analysis showing the repressive effect of miR-295 on FIH-1 expression. HK2 cells were treated as in panel E to collect lysate for immunoblot analysis. GAPDH was used as internal control in immunoblot analysis. (G) Quantification analysis of the related band intensity of FIH-1. (H) MicroRNA target reporter assay of FIH-1 3′-UTR. The putative miR-295 target sequence of the FIH-1 3′-UTR was cloned into the pMIR-REPORT vector. This and empty vector were transfected with miR-295 mimic or NC oligonucleotides to analyze luciferase activity. miR-295 mimic specifically reduced luciferase expression by pMIR-REPORT FIH-1 3′-UTR. All the quantitative data are expressed as mean±SD, *P < 0.05, **P < 0.01, ***P < 0.001, ****P < 0.0001. FIH-1, factor inhibiting hypoxia-inducible factor-1; UTR, untranslated region.

To understand how miR-295 contributes to HN, we investigated its downstream target genes. Bioinformatic analysis using online databases (TargetScan, http://www.targetscan.org) identified a putative miR-295 targeting sequence in the 3′-UTR of mouse FIH-1 mRNA, highly conserved across species (Figure 5A). To confirm FIH-1 as a miR-295 target, we assessed the effect of miR-295 on FIH-1 expression. Figure 5B shows that immunofluorescence staining revealed FIH-1 upregulation in renal tubule cells in HN, and miR-295 transfection significantly inhibited its expression. Immunoblot analysis further confirmed that miR-295 mimic transfection decreased FIH-1 expression in HN mice (Figure 5, C and D). Consistently, *in vitro* UA treatment induced FIH-1 in HK2 cells, and this induction was markedly suppressed by miR-295 mimic transfection (Figure 5E). The suppressive effect of miR-295 on FIH-1 was further verified by immunoblot analysis (Figure 5, F and G). To confirm direct targeting of FIH-1 by miR-295, we performed a luciferase reporter assay. The 3′-UTR sequence of FIH-1 was inserted into a luciferase reporter construct, which was cotransfected with either miR-295 mimic or NC oligonucleotides into HEK293 cells. As depicted in Figure 5H, cotransfection with miR-295 mimic (but not NC oligonucleotides) inhibited luciferase expression from the reporter construct containing the FIH-1 3′-UTR. These findings suggest that miR-295 directly targets FIH-1 mRNA to suppress its expression in HN.

### Knockdown of FIH-1 Inhibits UA-Induced Apoptosis and Profibrotic Cytokines Production in HK2 Cells

**Figure 6:**
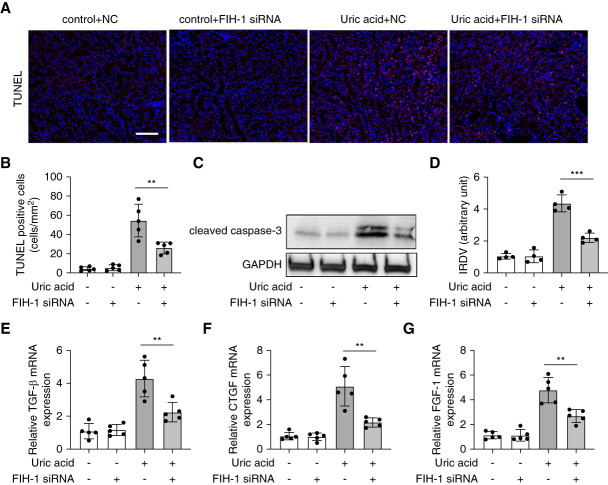
**Knockdown of FIH-1 inhibits UA-induced apoptosis and profibrotic cytokines production in HK2 cells.** HK2 cells were transfected with 40 nM FIH-1 siRNA or NC oligonucleotides and then treated with or without UA (800 *μ*M) for 36 hours. (A) Representative images of TUNEL staining, scale bar: 100 *μ*m. (B) Quantification of TUNEL-positive cells. (C) Immunoblot analysis of FIH-1, GAPDH was used as loading control. (D) Quantification analysis of the related FIH-1 band intensity, IRDV. (E–G) qPCR analysis for TGF-*β*, CTGF, and FGF-1, GAPDH was used as internal control. All the quantitative data are expressed as mean±SD, *P < 0.05, **P < 0.01, ***P < 0.001, ****P < 0.0001. CTGF, connective tissue growth factor.

Functionally, FIH-1 blocks the interaction of the HIF-1*α* with the transcriptional coactivator protein, leading to the inactivation of the HIF-1*α*.^28^ To determine FIH-1's role in HN, we investigated the effects of FIH-1 knockdown on apoptosis and profibrotic cytokine production in HK2 cells after UA treatment. As shown in Figure 6, A and B, TUNEL staining revealed that FIH-1 knockdown cells exhibited reduced apoptosis compared with cells transfected with control sequences. In addition, FIH-1 knockdown cells showed lower levels of caspase-3 activation after UA treatment, indicating decreased apoptosis (Figure 6, C and D). Furthermore, UA treatment induced the production of profibrotic cytokines, such as TGF-*β*, connective tissue growth factor, and FGF-1, and this induction was mitigated in FIH-1 knockdown cells (Figure 6, E–G). These results suggest that FIH-1 may contribute to tubular cell death and fibrosis in HN.

## Discussion

Clinically, the presence of hyperuricemia seems to increase the risk for developing CKD by two- to ten-fold. Importantly, some studies have suggested the risk for developing CKD is greater for hyperuricemic individuals than in participants with mild albuminuria.^29^ However, until now, the mechanisms of HN remain largely unclear, and effective treatment are still not available. In the present study, we have indicated that miR-295 was induced in renal tubular cells in an HIF-1*α*-dependent manner in HN.

Functionally, miR-295 induction inhibits tubular cell death and tubulointerstitial fibrosis in HN mouse models, indicating that miR-295 induction in HN is an adaptive or protective mechanism. Mechanistically, miR-295 may directly target and repress FIH-1, then promoting HIF-1*α* activation and finally affording a renal protective effect. Taking together, these findings unveil a potentially novel mechanism of HIF-1*α* regulation involving the positive feedback of miR-295 and FIH-1. Essentially, HIF-1*α* is activated in HN to promote the expression of miR-295, leading to the downregulation of its target gene FIH-1. As described above, FIH-1 could block the interaction of the HIF-1*α* with the transcriptional coactivator protein, leading to the inactivation of the HIF-1*α*.^28^ Thus, the downregulation of FIH-1 would promote HIF-1*α* activation, finally attenuating kidney injury and renal fibrosis.

In our previous study, we have found depleted HDAC3 could attenuate hyperuricemia-induced renal interstitial fibrosis through miR-19b-3p/SF3B3 axis.^25^ In another study, Du *et al*. indicated that miR-199a downregulation could alleviate HN through the PPAR*γ*/*β*-catenin axis.^30^ Thus, miRNAs have been implicated in the pathogenesis of HN, despite specific miRNAs involved in HN remain largely unknown. In this study, we demonstrated a significant increase of miR-295 in renal tubular cells after adenine and potassium oxalate oral administration in mice. Inhibition of miR-295 led to increased cell death and tubulointerstitial fibrosis after adenine and potassium oxalate treatment in mice, whereas supplementation of miR-295 mimic had kidney protective effects in this model. Thus, we have demonstrated compelling evidence to support a protective role of miR-295 in HN.

Importantly, our FISH analysis localized miR-295 induction mainly in renal tubule cells of the renal cortex. Tubular injury is widely recognized to be associated with the pathogenesis of renal fibrosis.^31^ Tubular epithelial cells are susceptible to TGF-*β*1, produced by both the injured tubule and the infiltrating cells, and instigate a fibrogenic program that includes epithelial-to-mesenchymal transition.^31^ In tubular epithelial cells, studies have indicated that sustained injury could activate HIF-1*α*.^31^ HIF-1*α* is a key regulator of cell responses to hypoxia, controlling a battery of genes that mediate adaptive responses against hypoxia.^32^ In addition, increasing evidence indicates that HIF-1*α* is a pivotal regulator of kidney fibrosis under various pathological conditions.^17^ However, it remains controversial whether HIF promotes or antagonizes renal fibrosis.^17^ In this study, we demonstrated HIF-1*α* activation in tubular epithelial cells in HN mice. Importantly, miR-295 induction by hyperuricemic was partly suppressed by YC-1, a pharmacological inhibitor of HIF-1*α*. Moreover, the chromatin immunoprecipitation assay demonstrated that HIF-1*α* bound to a specific site on the promoter of the miR-295 gene, and the binding was enhanced by UA treatment. Thus, HIF-1*α* could directly mediate miR-295 induction in HN. In fact, except for miR-295, studies also have indicated that HIF-1*α* could mediate the expression of many miRNAs in other kidney disease, such as miR-687, miR-688, and miR-1275.^33–35^

How does miR-295 contribute to HN? To address this, our *in vitro* and *in vivo* studies have identified FIH-1 as a direct target of miR-295. FIH-1 was identified as a protein that interacts with and inhibits the activity of HIF-1*α* in the *C*-terminal transactivation domain by coupling the oxidative decarboxylation of 2-oxoglutarate to the hydroxylation of HIF-1*α*.^36^ Studies have indicated that FIH-1 played a crucial role in the pathogenesis of CKD through targeting HIF-1*α*.^28^ In the kidney, HIF-1*α* is expressed by most renal tubular epithelial cells, and it is a critical molecule for mitigating hypoxia-induced damage and tubulointerstitial fibrosis.^37^ Despite HIF-1*α* remains controversial whether it promotes or antagonizes renal fibrosis, increasing studied have shown that enhancing HIF-1*α* signaling ameliorates the progression of renal fibrosis.^17^ For example, in ischemia-reperfusion injury, Kapitsinou *et al*. showed that activating HIF by pharmacological PHD inhibitor GSK1002083A before ischemia ameliorated AKI-induced fibrosis.^38^ In the model of hypertensive type 2 diabetes, pharmacological activating HIF by CoCl2 also attenuated renal fibrosis.^39^ In addition, Conde *et al*. found that HIF-1*α* siRNA increased the expression of fibrotic markers and promoted the epithelial-to-mesenchymal transition process in renal I/R.^40^ Of course, there are also some studies, like the one from Zhao *et al*. in 2021, have proposed a profibrotic role for HIF-1*α*.^16^ The divergence might be due to differences in experimental models, such as the type of kidney injury induction, animal models, or cell lines. In addition, the complex regulatory network of HIF-1*α* could contribute.^17,41^ In our study, knockdown of FIH-1 inhibits tubular cell apoptosis and profibrotic cytokines production in HK2 cells during UA treatment, suggesting an injurious role for FIH-1 in HN. Of course, reduced expression of FIH-1 means more activation of HIF-1*α*. Thus, we believe the renal protection of miR-295 is ultimately achieved by activating HIF-1*α*. These findings illustrate a positive feedback loop that enhance HIF-1*α* activation in renal tubular cells in HN.

However, there are several limitations to this study. First, although our results provide mechanistic insights from *in vivo* and *in vitro* experiments, we did not validate the findings in human renal specimens, which limits the direct clinical applicability of our conclusions. Second, this study was conducted exclusively in male mice to reduce the variability caused by sex hormone fluctuations. Considering the growing recognition of sex-based differences in immune and inflammatory responses, future investigations including both sexes and human samples will be necessary to confirm the generalizability of our findings.

In conclusion, this study demonstrates HIF-1*α*–mediated induction of miR-295 in renal tubular cells in HN. Notably, miR-295 induction leads to the downregulation of FIH-1, further enhancing the HIF-1*α* activation. Thus, this study unveils a HIF-1*α*/miR-295/FIH-1 positive feedback loop in HN which could enhance HIF-1*α* activation. This finding may provide a potential therapeutic target for HN.

## Supplementary Material

## Data Availability

All original data, including deidentified patient-level data or individual laboratory data measurements, are included in the manuscript and/or supplemental material.

## Declarative Statements

All animal experiments were conducted in accordance with the NIH Guide for the Care and Use of Laboratory Animals or an equivalent standard that meets or exceeds the ethical and welfare requirements outlined in the NIH Guide. All protocols were approved by the appropriate institutional animal care and use committee.

## Supplemental Material

Supplemental Figure 1. Changes in miR-295 expression in kidney tissues from diabetic kidney diseases mice.

Supplemental Figure 2. Successful delivery of miR-295 into the mice.

Supplemental Figure 3. Successful delivery of anti-miR-295 locked nucleic acid into the mice.
