# Supplementary material for: The Positive Feedback Loop of Hypoxia-Inducible Factor-1α/miR-295/Factor Inhibiting Hypoxia-Inducible Factor-1 in Hyperuricemic Nephropathy
Source: Kidney360. 2025 Dec 23;7(4):741–53. doi: 10.34067/KID.0000001069 (PMC13134801; doi:10.34067/KID.0000001069)
Supplement: Supplementary file 2 [file kidney360-7-741-s002.pdf]

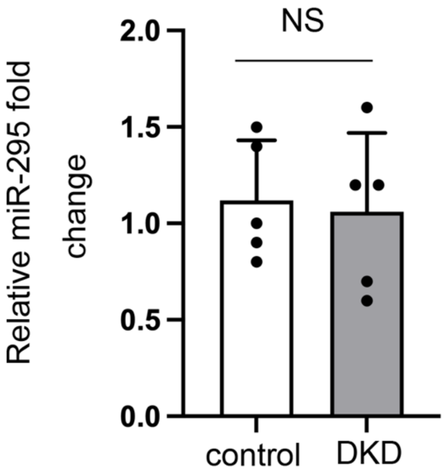

Supplemental Figure 1: Changes in miR-295 expression in kidney tissues from Diabetic Kidney Diseases (DKD) mice. qPCR analysis showing miR-295 was not changed in DKD. Eight-week-old C57BL/6J mice were injected with 50 mg/kg STZ for 5 consecutive days. Control mice were injected with normal saline. The mice were euthanized after 12 weeks. Quantitative data are expressed as mean ± SD, n=5, NS represents not statistically significant.

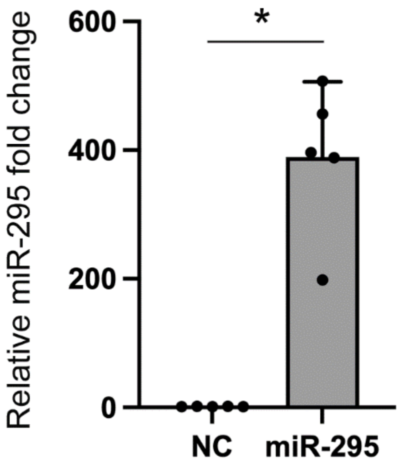

Supplemental Figure 2: Successful delivery of miR-295 into the mice. miR-295 mimic (3mg/kg) or NC oligonucleotide LNA were delivered to mice through tail vein injection every 2 days after PO injection. qPCR analysis of miR-295 in mouse kidneys. The level of miR-295 was normalized to the level of U6 (internal loading control) of the same samples to determine the ratio with the ratio of control mice arbitrarily set as 1. All the values are expressed as mean ± SD (n = 5), $*p < 0.05$.

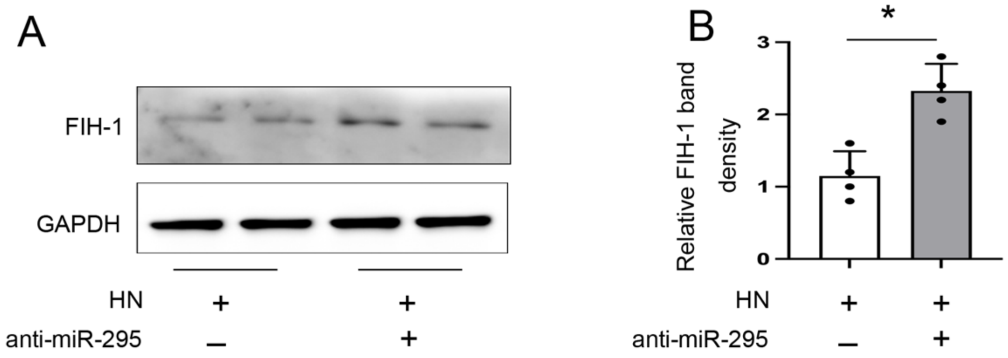

Supplemental Figure 3: Successful delivery of anti-miR-295 LNA into the mice. HN was induced by potassium oxonate (PO) intraperitoneally and adenine (Ad) orally administered for two weeks. Except for the control group, each group was intraperitoneally administered PO (350 mg/kg/d) and orally administered with Ad (70 mg/kg/d) to induce HN at 8:30 am for 14 consecutive days to induce HN. Control mice were treated with normal saline. At the end of 21st day, the animals were sacrificed. Anti-miR-295 LNA (6 mg/kg) or NC oligonucleotide LNA were delivered to mice through tail vein injection every 2 days after PO injection.**(A)**Expressions of FIH-1 was detected by Western blot; GAPDH was used as internal control; **(B)** Quantification analysis of the related band intensity of FIH-1, data are expressed as mean ± SD (n = 4).
